# Early-Life Bisphenol A Exposure and Child Body Mass Index: A Prospective Cohort Study

**DOI:** 10.1289/ehp.1408258

**Published:** 2014-07-29

**Authors:** Joseph M. Braun, Bruce P. Lanphear, Antonia M. Calafat, Sirad Deria, Jane Khoury, Chanelle J. Howe, Scott A. Venners

**Affiliations:** 1 Department of Epidemiology, Brown University School of Public Health, Brown University, Providence, Rhode Island, USA; 2 Child and Family Research Institute, BC Children’s and Women’s Hospital, Vancouver, Canada; 3 Faculty of Health Sciences, Simon Fraser University, Burnaby, British Columbia, Canada; 4 Centers for Disease Control and Prevention, Atlanta, Georgia, USA; 5 Division of Biostatistics and Epidemiology, Cincinnati Children’s Hospital Medical Center, Cincinnati, Ohio, USA

## Abstract

Background: Early-life exposure to bisphenol A (BPA) may increase childhood obesity risk, but few prospective epidemiological studies have investigated this relationship.

Objective: We sought to determine whether early-life exposure to BPA was associated with increased body mass index (BMI) at 2–5 years of age in 297 mother–child pairs from Cincinnati, Ohio (HOME Study).

Methods: Urinary BPA concentrations were measured in samples collected from pregnant women during the second and third trimesters and their children at 1 and 2 years of age. BMI *z*-scores were calculated from weight/height measures conducted annually from 2 through 5 years of age. We used linear mixed models to estimate BMI differences or trajectories with increasing creatinine-normalized BPA concentrations.

Results: After confounder adjustment, each 10-fold increase in prenatal (β = –0.1; 95% CI: –0.5, 0.3) or early-childhood (β = –0.2; 95% CI: –0.6, 0.1) BPA concentrations was associated with a modest and nonsignificant reduction in child BMI. These inverse associations were suggestively stronger in girls than in boys [prenatal effect measure modification (EMM) *p*-value = 0.30, early-childhood EMM *p*-value = 0.05], but sex-specific associations were imprecise. Children in the highest early-childhood BPA tercile had lower BMI at 2 years (difference = –0.3; 95% CI: –0.6, 0.0) and larger increases in their BMI slope from 2 through 5 years (BMI increase per year = 0.12; 95% CI: 0.07, 0.18) than children in the lowest tercile (BMI increase per year = 0.07; 95% CI: 0.01, 0.13). All associations were attenuated without creatinine normalization.

Conclusions: Prenatal and early-childhood BPA exposures were not associated with increased BMI at 2–5 years of age, but higher early-childhood BPA exposures were associated with accelerated growth during this period.

Citation: Braun JM, Lanphear BP, Calafat AM, Deria S, Khoury J, Howe CJ, Venners SA. 2014. Early-life bisphenol A exposure and child body mass index: a prospective cohort study. Environ Health Perspect 122:1239–1245; http://dx.doi.org/10.1289/ehp.1408258

## Introduction

Child obesity is one of the greatest public health challenges worldwide (World Health Organization 2010). Excess food consumption and inadequate physical activity are major risk factors for obesity, but emerging evidence suggests that exposure to obesogens—chemicals that alter adipogenesis or metabolism—might play a role in increasing obesity risk beyond these traditional risk factors (Janesick and Blumberg 2012; Romano et al. 2014; Tang-Peronard et al. 2011). The developing fetus and infant may be especially sensitive to obesogens because of their immature detoxification pathways and sensitivity to environment inputs. Most epidemiological studies of environmental chemical obesogens have been limited to organochlorine compounds; few have examined contemporary chemicals, such as bisphenol A (BPA) (Tang-Peronard et al. 2011).

BPA is a high-production-volume chemical used to produce polycarbonate plastics and resins, and there is ubiquitous exposure among persons in industrialized countries (Braun et al. 2012; Lee et al. 2014; Quirós-Alcalá et al. 2013; Valvi et al. 2013). BPA is a suspected endocrine disruptor and may affect the metabolism or action of hormones or receptors involved in the etiology of obesity, including glucocorticoids, gonadal hormones, and peroxisome proliferator activated receptors (Janesick and Blumberg 2012; Ross and Desai 2013). One animal study suggests that the obesogenic effect of BPA may by modified by the availability of methyl donors (e.g., folate) for DNA methylation, thus permanently altering the programming of adipogenesis, appetite, or energy metabolism, and increasing later-life obesity risk (Dolinoy et al. 2007).

Although some animal studies suggest that BPA is a candidate obesogen, others do not (reviewed by Harley et al. 2013). Cross-sectional human studies suggest that urinary BPA concentrations are associated with increased body mass index (BMI) or obesity in adults and children, but these findings could result from confounding or reverse causation because diet is an important source of BPA exposure and obesity is linked to certain dietary patterns (Carwile and Michels 2011; Sharpe and Drake 2013; Trasande et al. 2012; Wang et al. 2012). Two prospective cohort studies examining early-life BPA exposure report contradictory findings: One found higher BMI among children with higher prenatal BPA exposure (Valvi et al. 2013), and another reported lower BMI with higher prenatal exposure (Harley et al. 2013). These studies suggest that girls, as well as children born to women who smoke during pregnancy, may be more susceptible to prenatal BPA exposure.

We investigated whether prenatal or early-childhood BPA exposure was associated with BMI or waist circumference in children 2–5 years of age from a population-based, prospective cohort study conducted in Cincinnati, Ohio. We also determined whether the association between prenatal BPA exposure and child BMI was modified by maternal folate levels, child sex, or prenatal tobacco smoke exposure.

## Methods

*Study participants*. We used data from the Health Outcomes and Measures of the Environment (HOME) Study, a prospective cohort study designed to examine the health impact of early-life exposure to prevalent environmental chemicals (Dietrich et al. 2005). We recruited pregnant women from nine prenatal clinics associated with three hospitals in the Cincinnati area from March 2003 through January 2006. Eligibility criteria and enrollment have been previously described (Braun et al. 2009). All women provided written informed consent for themselves and their children after the study protocols had been explained. The institutional review boards of Cincinnati Children’s Hospital Medical Center, the cooperating delivery hospitals, and the Centers for Disease Control and Prevention (CDC) approved this study.

*Maternal and child BPA exposure assessment*. Because there is concern that BPA exposures may adversely affect child health depending on the timing of exposure, we examined exposures during two distinct periods of development—prenatal and early childhood. Women provided up to two spot urine samples in polypropylene cups at their prenatal care clinic visits around 16 and 26 weeks of pregnancy. Children provided up to two spot urine samples at annual clinic or home visits when they were around 1 and 2 years of age (see Supplemental Material, Table S1, for means and ranges). If a child did not provide a sample at the 1- or 2-year clinic visits, we used urine samples collected during home visits. Before urine collection, each child’s genital area was wiped with a Wet-Nap (http://wetnap.com) by their caregiver. For children who were not toilet trained, we placed a surgical insert into a clean diaper at the beginning of the study visit and checked the diaper for urine at the end of the study visit. If the diaper was wet and free of stool, the insert was placed into a polyethylene urine collection cup, and urine was expressed from the insert with a syringe. For children who were being toilet trained, a training toilet was lined with inserts. For toilet-trained children, urine samples were collected directly into a urine collection cup with the aid of the child’s caregiver. All samples were refrigerated until they were processed, after which they were stored at or below –20^o^C until shipped on dry ice to CDC for analysis. BPA concentrations were measured at the CDC National Center for Environmental Health laboratories using previously described analytic chemistry methods (Ye et al. 2008). In 2009, we found nondetectable (< 0.4 ng/mL) levels of BPA in surgical inserts and wipes used to collect child urine.

To account for urine dilution, urinary creatinine was measured by a kinetic Jaffe reaction, and BPA concentrations were divided by creatinine and multiplied by 100 to yield units of micrograms BPA per gram creatinine.

We averaged log_10_-transformed maternal and child creatinine-normalized BPA concentrations to create prenatal and early-childhood BPA exposure measures, respectively. The prenatal exposure measure used maternal urinary BPA concentrations at 16 and 26 weeks gestation (7 had one measure and 290 had two measures). The early-childhood exposure measure used child urinary BPA concentrations at 1 and 2 years of age (90 had one measure and 195 had two measures). We characterized creatinine-normalized urinary BPA concentrations as terciles or continuous log_10_-transformed values in our statistical models.

*Child anthropometry*. Weight, height, and waist circumference were measured in triplicate and averaged at each annual study visit. We obtained child’s weight at 2–5 years to the nearest 0.01 kg, with the child dressed in undergarments or a dry diaper, using a ScaleTronix scale (White Plains, NY). If the child was uncooperative, we obtained a sitting weight using a ScaleTronix Pediatric Scale Model 4802. Height at 2–5 years was measured to the nearest 0.1 cm using an Ayrton Stadiometer Model S100 with the child standing straight without shoes or head coverings and heels positioned against the wall. If the child had a hairstyle that prevented the child’s head from lying flush against the head board, the height of the hairstyle was subtracted from the height measure. Waist circumference was measured at 4 and 5 years of age by placing a plastic measuring tape around a horizontal plane defined by the left and right iliac crests. Child BMI was converted to age- and sex-specific *z*-scores using U.S. references available from the National Center for Health Statistics (Kuczmarski et al. 2000). Research staff who conducted anthropometric measures were blinded to children’s urinary BPA concentrations.

*Confounding variables*. We considered adjusting for potential confounders that might be associated with both BPA exposure and growth/size. Trained research assistants collected sociodemographic, perinatal, and dietary/activity variables using standardized computer-assisted interviews and medical chart reviews. Sociodemographic covariates included maternal race, age, education, marital status, household income, insurance status, and food security during pregnancy. Perinatal variables included maternal depressive symptoms at 16 weeks gestation (Beck Depression Inventory-II) (Beck et al. 1996), BMI at 16 weeks gestation, parity, and serum cotinine (a sensitive and specific biomarker of tobacco smoke exposure) (Braun et al. 2010).

Our dietary questions were originally designed to assess environmental chemical exposures (e.g., organophosphate pesticides), not macro- or micronutrient intake. We adjusted for frequency of maternal or child canned vegetable and fresh fruit/vegetable consumption because we previously found that canned vegetable consumption was associated with higher maternal urinary BPA concentrations and may be associated with diet quality (Braun et al. 2011). Dietary variables were collected during pregnancy for mothers and annually at 2–5 years of age for children. We adjusted for prenatal vitamin use and breastfeeding duration because vitamins may be a source of methyl donors, and breastfeeding may decrease child obesity risk, respectively (Anderson et al. 2012; Lefebvre and John 2014). Child activity variables were collected annually at 2–5 years of age and included parent-reported hours of daily television watching and outdoor time.

We created unadjusted and several sets of adjusted models to verify the robustness of our results to potential confounding and selection bias. We created a primary model adjusting for sociodemographic and perinatal variables, and then additionally adjusted for maternal nutrition, child nutrition, child activity, child age, or maternal/child urinary di(2-ethylhexyl) phthalate (DEHP) metabolite concentrations. Urinary DEHP concentrations were measured using previously described methods (Silva et al. 2007). We also adjusted for both prenatal and early-childhood urinary BPA concentrations simultaneously in the same model.

*Statistical analyses*. We began by describing the univariate characteristics of urinary BPA concentrations and calculating Pearson correlation coefficients between log_10_-transformed concentrations. Next, we calculated the mean BMI *z*-score at each age, as well as the number and percent of children with BMI *z*-scores ≥ the 85th percentile (overweight). We then tabulated the mean BMI *z*-scores and median urinary BPA concentrations according to covariates.

We examined whether higher prenatal or early-childhood BPA concentrations were associated with differences in BMI *z*-scores at 2–5 years or waist circumference at 4 and 5 years using a linear mixed model with an unstructured correlation matrix, random intercept, and empirical standard errors. This model accounts for the repeated and correlated measurements within an individual and increases our statistical precision by borrowing information across repeated measures (Fitzmaurice et al. 2004). The unstructured covariance matrix produced the best model fit according to the Akaike Information Criterion compared to compound symmetric or autoregressive covariance matrices. The coefficients from this model can be interpreted as the mean difference in BMI *z*-score averaged across 2–5 years of age with increasing BPA concentrations.

Then we used this same model to examine children’s BMI *z*-score slopes between 2 and 5 years of age according to BPA concentration terciles. We modeled BMI *z*-scores as a function of BPA tercile, child age in months, an interaction term between age and BPA tercile, and covariates. This model allows each BPA tercile to have its own linear BMI *z*-score slope over time (i.e., 2–5 years of age). We then estimated the BMI slope per year for each BPA tercile and determined whether these slopes were statistically different from one another using the age × BPA interaction terms.

We calculated the odds of being overweight (BMI *z*-score ≥ 85th percentile) according to BPA concentration using generalized linear mixed models with an unstructured correlation matrix and random intercept. Finally, we examined whether the associations between BPA concentrations and BMI differed in boys and girls.

*Secondary analyses*. On the basis of prior studies examining prenatal BPA or other environmental chemical exposures and infant/child growth, we used product effect measure modification (EMM) terms to examine whether the association between prenatal urinary BPA concentrations and BMI was modified by prenatal tobacco smoke exposure, prenatal whole blood folate levels, and maternal race (Dolinoy et al. 2007; Rauch et al. 2012; Valvi et al. 2013). We classified women as smokers if they had serum cotinine levels ≥ 3 ng/mL at 16 or 26 weeks gestation or birth; otherwise they were classified as nonsmokers (Benowitz et al. 2009). Whole-blood folate levels were measured in samples collected at 16 weeks gestation using previously described methods (Fazili and Pfeiffer 2004). We classified women into terciles based on the distribution of whole blood folate concentrations (34–387, 392–597, and 599–1,660 nmol/L). We only examined whites and blacks when examining EMM by race given the small sample size in the “Other” race group.

We also conducted analyses excluding infants born small for gestational age (weight for gestational age < 10th percentile), women with gestational diabetes or hypertension, and one woman with a 26-week BPA concentration about 600 times higher than the median BPA concentration to determine whether including these participants unduly influenced our results (Sathyanarayana et al. 2011). We re-ran our primary analyses without creatinine-normalizing urinary BPA concentrations. Finally, we examined the cross-sectional associations between children’s urinary BPA concentrations and BMI at 2–5 years of age. Urinary BPA concentrations were measured in urine samples collected at 3, 4, and 5 years of age using the methods described above.

## Results

Of 389 women who gave birth to singleton infants, 297 (76%) with complete prenatal exposure data and covariates returned to our study clinic at least once for a total of 889 study visits during child’s age 2–5 years (285 for early-childhood exposure analyses, 73%, 864 visits).

**Figure 1:**
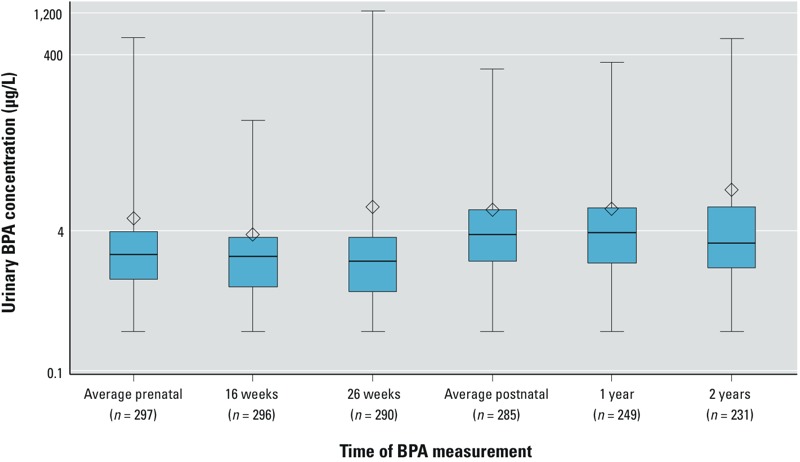
Urinary BPA concentrations during pregnancy at 16 and 26 weeks and the first 2 years of life among Cincinnati, Ohio, women and their children. Whiskers represent the minimum and maximum, box edges represent the 25th and 75th percentiles, the line in the box represents the median, and the diamond represents the arithmetic mean. The number of mothers/children for the average concentrations is greater than the individual concentrations because not all the same participants returned at the both visits.

Median urinary BPA concentrations were lower in women than in their children (Figure 1, Table 1; see also Supplemental Material, Table S2). Creatinine-normalized BPA concentrations at 16 and 26 weeks (Pearson *r* = 0.09) or 1 and 2 years (Pearson *r* = 0.10) were not correlated; however non-normalized concentrations were weakly correlated (Pearson *r* ≤ 0.3; see Supplemental Material, Table S3). Averaged creatinine-normalized maternal urinary BPA concentrations were not correlated with averaged children’s concentrations (Pearson *r* = 0.03, *p* = 0.67), but non-normalized concentrations were weakly correlated (Pearson *r* = 0.17, *p* < 0.01).

**Table 1:** Urinary BPA concentrations and child BMI *z*-scores according to maternal and child covariates among Cincinnati, Ohio, women and their children.

Covariate	*n*	Maternal median BPA μg/L (25th, 75th)^*a*^	Child median BPA μg/L (25th, 75th)^*b*^	BMI *z*-score (mean ± SD)^*c*^
Overall	297	2.1 (1.1, 3.9)	3.6 (1.8, 6.9)	0.04 ± 1.04
Maternal race
White	198	1.7 (0.9, 2.9)	2.7 (1.5, 5.4)	0.03 ± 1.00
Black	82	3.9 (2.4, 6.3)	5.5 (3.4, 9.6)	0.10 ± 1.15
Other	17	2.3 (1.7, 3.2)	4.7 (2.6, 6.2)	–0.06 ± 1.00
Maternal age (years) at delivery
< 25	58	3.5 (1.9, 6.2)	4.9 (2.5, 7.6)	0.05 ± 1.16
25 to < 35	192	1.9 (1.0, 3.7)	3.4 (1.6, 6.2)	0.04 ± 1.02
≥ 35	47	1.7 (0.7, 3.2)	3.1 (1.7, 7.6)	0.04 ± 1.01
Maternal education
Graduate/professional/bachelor	166	1.7 (0.8, 2.7)	2.6 (1.5, 5.3)	0.08 ± 0.90
Some college	74	2.8 (1.7, 4.8)	4.2 (2.6, 7.4)	–0.15 ± 1.08
High school	31	3.2 (2.1, 6.2)	4.9 (2.5, 7.1)	0.39 ± 1.04
< High school	26	6.1 (3.2, 7.5)	5.9 (3.5, 12.0)	–0.10 ± 1.56
Marital status
Married	207	1.8 (0.9, 2.9)	2.9 (1.6, 5.8)	0.05 ± 1.01
Unmarried, living together	33	3.2 (2.2, 6.4)	4.2 (2.3, 7.3)	0.26 ± 1.00
Unmarried, living alone	57	3.7 (1.9, 6.3)	5.2 (3.5, 9.4)	–0.10 ± 1.16
Household income (per year)
≥ $80,000	88	1.6 (0.8, 2.6)	2.9 (1.5, 5.3)	0.01 ± 0.87
$40,000–80,000	110	1.8 (0.9, 3.0)	2.7 (1.6, 5.7)	0.12 ± 1.10
$20,000–$40,000	41	2.9 (1.8, 4.5)	4.3 (2.9, 8.0)	–0.06 ± 1.14
< $20,000	58	5.1 (2.6, 7.5)	5.3 (3.2, 9.2)	0.01 ± 1.10
Maternal employment
No	52	2.3 (1.4, 6.2)	3.9 (2.0, 8.2)	–0.07 ± 1.06
Yes	245	2.0 (1.0, 3.8)	3.4 (1.7, 6.7)	0.07 ± 1.04
Maternal insurance
Private	226	1.8 (0.9, 3.0)	3.0 (1.6, 5.9)	0.06 ± 1.00
Public/none	71	4.3 (2.3, 6.9)	5.0 (3.1, 9.1)	–0.02 ± 1.15
Maternal depressive symptoms
Minimal	245	2.0 (1.0, 3.8)	3.5 (1.8, 6.7)	–0.01 ± 1.02
Mild	32	2.8 (1.8, 6.3)	4.0 (1.7, 9.1)	0.21 ± 1.02
Moderate/severe	20	2.7 (1.3, 4.3)	3.7 (2.1, 7.0)	0.41 ± 1.25
Maternal serum cotinine concentration (ng/mL)
Unexposed (< 0.015)	120	1.5 (0.8, 2.7)	2.7 (1.4, 6.2)	–0.04 ± 0.98
Secondhand (0.015–3)	150	2.7 (1.5, 4.4)	3.9 (2.3, 7.3)	0.09 ± 1.07
Active (≥ 3)	27	4.2 (2.3, 6.7)	3.7 (1.7, 6.2)	0.16 ± 1.14
Maternal BMI (kg/m^2^)
< 25	126	1.9 (1.0, 3.5)	3.4 (1.8, 7.1)	–0.14 ± 1.02
25 to < 30	101	1.7 (0.8, 3.2)	2.8 (1.7, 5.4)	0.05 ± 0.94
≥ 30	70	3.4 (2.1, 6.2)	4.7 (2.3, 9.4)	0.35 ± 1.16
Parity
0	128	1.8 (0.8, 3.7)	3.3 (1.6, 5.6)	0.03 ± 1.00
1	98	2.2 (1.4, 4.1)	3.7 (2.0, 7.3)	0.14 ± 0.99
≥ 2	71	2.4 (1.5, 4.2)	4.0 (2.0, 8.2)	–0.07 ± 1.17
Prenatal vitamin use
Never or few times/month	41	2.7 (1.8, 5.1)	4.5 (3.1, 8.2)	–0.02 ± 1.22
Daily or weekly	256	2.0 (1.0, 3.8)	3.4 (1.7, 6.3)	0.05 ± 1.01
Prenatal canned vegetable consumption frequency
≤ Monthly	83	1.8 (0.8, 3.8)	3.3 (1.8, 5.6)	0.08 ± 0.90
Weekly	174	2.1 (1.2, 4.0)	3.5 (1.6, 6.7)	0.06 ± 1.07
≥ Daily	40	2.1 (1.4, 3.9)	5.4 (3.0, 9.3)	–0.13 ± 1.18
Prenatal fresh fruit and vegetable consumption frequency
Monthly/weekly	175	2.4 (1.3, 4.2)	3.8 (2.0, 7.6)	0.03 ± 1.06
≥ Daily	122	1.8 (0.9, 3.5)	3.2 (1.6, 5.7)	0.05 ± 1.01
Breastfeeding duration (months)
None	49	2.8 (1.5, 6.0)	2.9 (1.8, 8.2)	–0.02 ± 1.19
> 0 to 3.25	83	2.9 (1.7, 4.8)	4.0 (2.3, 6.5)	0.10 ± 1.15
3.5 to 10.5	80	1.8 (0.9, 3.2)	3.2 (1.6, 6.2)	0.09 ± 0.95
> 10.5	85	1.9 (0.9, 3.0)	3.4 (1.8, 7.3)	–0.03 ± 0.93
Child canned vegetable consumption frequency
≤ Monthly	47	1.9 (0.9, 3.5)	2.6 (1.5, 5.1)	0.14 ± 0.82
Weekly	192	2.1 (1.1, 3.9)	3.6 (1.8, 7.3)	0.03 ± 1.08
≥ Daily	49	2.3 (0.9, 3.9)	3.8 (2.3, 6.3)	–0.04 ± 1.07
Child fresh fruit and vegetable consumption frequency
Monthly/weekly	149	2.6 (1.3, 4.3)	4.2 (2.1, 8.1)	–0.01 ± 1.07
≥ Daily	139	1.8 (0.9, 3.3)	2.7 (1.5, 5.2)	0.08 ± 1.01
Child daily TV watching (hr)
< 1	123	2.0 (1.0, 3.5)	3.4 (1.7, 5.6)	0.06 ± 0.99
1–2	85	1.9 (1.0, 3.8)	3.1 (1.5, 7.8)	0.00 ± 1.09
> 2	78	2.7 (1.5, 5.1)	4.0 (2.3, 7.5)	0.07 ± 1.07
Child daily outdoor time (hr)
< 1	204	2.2 (1.1, 4.1)	3.8 (1.8, 7.3)	0.07 ± 1.11
1–2	44	2.0 (0.9, 3.8)	3.3 (1.6, 6.2)	–0.08 ± 0.88
> 2	40	1.6 (0.9, 3.2)	2.9 (2.0, 4.6)	0.02 ± 0.82
25th and 75th are percentiles. ^***a***^Average BPA concentration in maternal 16- and 26-week gestation urine samples (*n *= 297). ^***b***^Average BPA concentration in child 1- and 2-year urine samples (*n *= 285). ^***c***^BMI *z*-score at the child’s first visit, if more than one was available.				

Children’s BMI *z*-scores ranged from a mean of 0 to 0.2 standard deviation scores (SDS) between 2 and 5 years of age; 16–19% of children had BMI *z*-scores ≥ the 85th percentile (Supplemental Material, Figure S1).

Higher prenatal urinary BPA concentrations were observed in mothers who were black, were younger at delivery, had less household income and education, consumed more canned vegetables and fewer fresh fruits and vegetables, or breastfed for a shorter duration (Table 1). Similar patterns were observed for early-childhood urinary BPA concentrations.

After confounder adjustment, higher prenatal or early-childhood urinary BPA concentrations were not associated with BMI *z*-scores in children at 2–5 years of age (Table 2). Results were similar regardless of adjustment for dietary/activity factors, child age, or both BPA exposures (see Supplemental Material, Table S4). Not normalizing BPA concentrations for creatinine attenuated both the prenatal and early-childhood estimates to null (see Supplemental Material, Table S5). Both prenatal and early-childhood urinary BPA concentrations were associated with smaller waist circumference at 4 and 5 years of age, but the 95% confidence intervals (CI) of the point estimates included the null value (see Supplemental Material, Table S6).

**Table 2:** Adjusted change in child BMI *z*-score between 2–5 years of age (β) by tercile of or with a 10-fold increase in maternal or early childhood urinary BPA concentrations among Cincinnati, Ohio, women and their children.^*a*^

BPA exposure measure	*n*	Mean BMI *z*-score	β (95% CI)	*p*-Value
Prenatal
1st tercile (0.4–1.6 μg/g creatinine)	99	0.00	Referent	Referent
2nd tercile (1.6–2.6 μg/g creatinine)	99	–0.01	0.0 (–0.3, 0.3)	0.98
3rd tercile (2.6–49 μg/g creatinine)	99	0.05	0.1 (–0.2, 0.3)	0.66
Continuous, log_10_-transformed	297		–0.1 (–0.5, 0.3)	0.51
Early childhood
1st tercile (2.1–11 μg/g creatinine)	95	0.13	Referent	Referent
2nd tercile (11–20 μg/g creatinine)	95	0.12	0.0 (–0.3, 0.3)	0.96
3rd tercile (20–314 μg/g creatinine)	95	–0.10	–0.2 (–0.5, 0.1)	0.12
Continuous, log_10_-transformed	285		–0.2 (–0.6, 0.1)	0.19
^***a***^Adjusted for maternal race (white, black, and other), marital status (married living together, unmarried living together, and unmarried living alone), parity (0, 1, and ≥ 2), age at delivery (continuous, years), household income (continuous, $10,000 increments), education (< high school, high school, some college, and ≥ bachelor’s degree), employment (any and none), insurance (private and public/none), BMI at 16 weeks of pregnancy (continuous, kg/m^2^), depressive symptoms at baseline (continuous), and prenatal serum cotinine (continuous, log_1_-transformed).				

**Figure 2:**
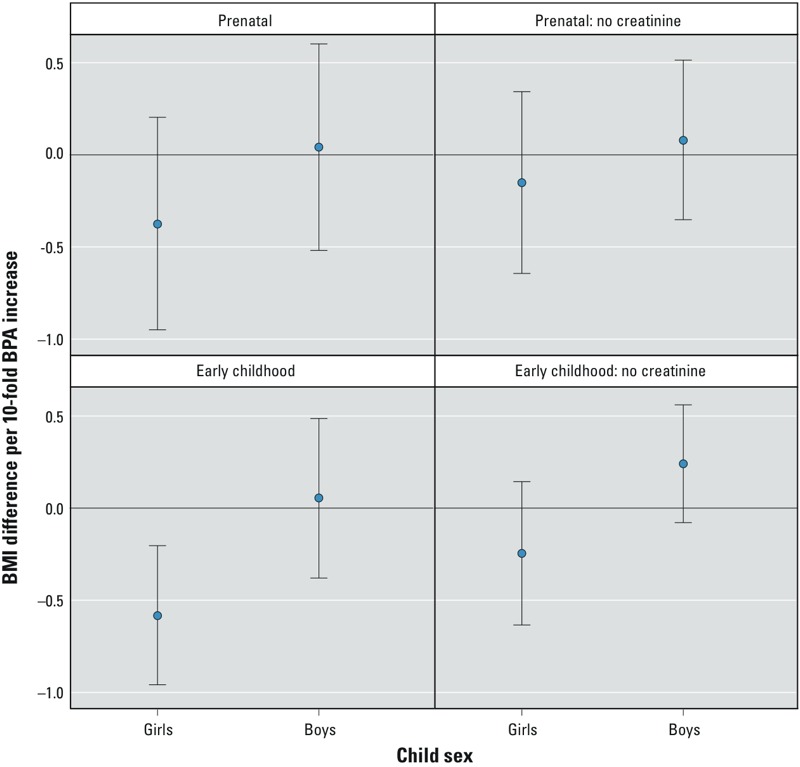
Adjusted change in child BMI *z*-score between 2 and 5 years of age with a 10-fold increase in maternal or early-childhood urinary BPA concentrations among Cincinnati, Ohio, women and their children, stratified by child sex. Adjusted for maternal race (white, black, and other), marital status (married living together, unmarried living together, and unmarried living alone), parity (0, 1, ≥ 2), age at delivery (continuous, years), household income (continuous, $10,000 increments), education (< high school, high school, some college, and ≥ bachelor’s degree), employment (any and none), insurance (private and public/none), BMI at 16 weeks (continuous, kg/m^2^), depressive symptoms at baseline (continuous), and prenatal serum cotinine (continuous, log_10_-transformed). Prenatal effect-measure modification *p*-values: with creatinine: 0.30; no creatinine: 0.39. Early-childhood effect-measure modification *p*-values: with creatinine: 0.05; no creatinine: 0.06. Error bars represent 95% CIs.

Inverse associations between maternal urinary BPA concentrations and child BMI were slightly stronger among girls (β = –0.4; 95% CI: –0.9, 0.2; *n* = 165) compared with boys (β = 0.0; 95% CI: –0.5, 0.6; *n* = 132), although the EMM *p*-value did not reach conventional levels of significance (*p* = 0.30) (Figure 2). The evidence for EMM was stronger for early-childhood urinary BPA concentrations (*p* = 0.05), where higher concentrations were associated with lower child BMI among girls (β = –0.6; 95% CI: –1.1, –0.1; *n* = 155) than among boys (β = 0.1; 95% CI: –0.4, 0.5; *n* = 130). The magnitude of the differences between the sexes was attenuated when BPA concentrations were not creatinine-normalized (see Supplemental Material, Table S7).

Each 10-fold increase in maternal urinary BPA concentrations was associated with a modestly decreased odds of being overweight between 2 and 5 years of age [odds ratio (OR) = 0.65; 95% CI: 0.19, 2.18; *p* = 0.48], but the OR CI included the null value. The association between early-childhood urinary BPA concentrations and being overweight was much closer to null (OR = 0.93; 95% CI: 0.34, 2.53; *p* = 0.89).

**Figure 3:**
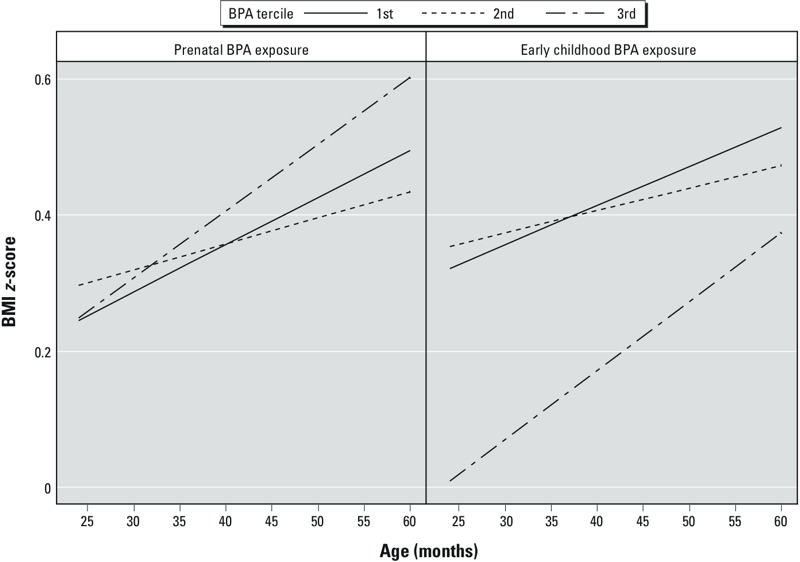
Adjusted BMI *z*-scores slopes between 2 and 5 years of age by prenatal and early-childhood BPA tercile among Cincinnati, Ohio, women and their children. Adjusted for maternal race (white, black, and other), marital status (married living together, unmarried living together, and unmarried living alone), parity (0, 1, ≥ 2), age at delivery (continuous, years), household income (continuous, $10,000 increments), education (< high school, high school, some college, and ≥ bachelor’s degree), employment (any and none), insurance (private and public/none), BMI at 16 weeks (continuous, kg/m^2^), depressive symptoms at baseline (continuous), and prenatal serum cotinine (continuous, log_10_-transformed). Prenatal BPA terciles: 1st tercile: 0.4–1.6 μg/g creatinine; 2nd tercile: 1.6–2.6 μg/g creatinine; and 3rd tercile: 2.6–49 μg/g creatinine. Early-childhood BPA terciles: 1st tercile: 2.1–11 μg/g creatinine; 2nd tercile: 11–20 μg/g creatinine; and 3rd tercile: 20–314 μg/g creatinine. Prenatal BPA × age interaction *p*-values: 2nd vs. 1st tercile: 0.42; 3rd vs. 1st tercile: 0.43. Early-childhood BPA × age interaction *p*-values: 2nd vs. 1st tercile: 0.51; 3rd vs. 1st tercile: 0.22.

There was not strong evidence that maternal urinary BPA concentrations were positively associated with rapid growth between 2 and 5 years of age (Figure 3) (age × BPA interaction term *p*-value = 0.26). There was stronger evidence that BMI slopes increased more rapidly between 2 and 5 years among children in the highest tercile of early-childhood BPA concentrations (BMI increase per year = 0.12; 95% CI: 0.07, 0.18) compared with children in the first (BMI increase per year = 0.07; 95% CI: 0.01, 0.13) or second (BMI increase per year = 0.04; 95% CI: –0.02, 0.11) terciles (age × BPA tercile interaction *p*-value = 0.14). This increase was coincident with lower BMI at 2 years of age among children in the third tercile compared with children in the first tercile (BMI difference = –0.3; 95% CI: –0.6, 0.0), though BMI differences were not evident at 5 years of age (BMI difference = –0.1; 95% CI: –0.5, 0.2). BMI slopes no longer differed when early-childhood BPA concentrations were not creatinine-normalized (first tercile = 0.04; 95% CI: –0.02, 0.10; second tercile = 0.09; 95% CI: 0.04, 0.15; third tercile = 0.09; 95% CI: 0.03, 0.15; *p*-value for interaction = 0.42).

There was not strong evidence that the associations between prenatal or early-childhood BPA concentrations and BMI *z*-score slopes differed according to child sex (EMM *p*-values = 0.18 to 0.80). However, we had a relatively small number of children for this analysis (see Supplemental Material, Figures S2 and S3).

*Secondary analyses*. Associations between continuous prenatal urinary BPA concentrations and child BMI did not differ (EMM *p*-value = 0.98) among children born to mothers who smoked during pregnancy (β = –0.1; 95% CI: –1.9, 1.6, *n* = 29) compared with those with mothers who did not smoke (β = –0.1; 95% CI: –0.5, 0.3, *n* = 268). The associations between prenatal urinary BPA concentrations and child BMI did not differ according to terciles of maternal whole-blood folate levels (EMM *p* = 0.74). There was no evidence that associations between prenatal or early-childhood urinary BPA concentrations and BMI differed between blacks or whites (EMM *p*-values > 0.34). Our results did not change appreciably when we adjusted for children’s serum cotinine levels, adjusted for maternal or child urinary DEHP concentrations, or excluded infants born small for gestational age, women with gestational diabetes or pregnancy-induced hypertension, or the woman with the exceptionally high BPA concentration (see Supplemental Material, Table S5). The cross-sectional associations between children’s concurrent urinary BPA concentrations and BMI *z*-scores at 2–5 years were both positive and negative in direction (see Supplemental Material, Table S8).

## Discussion

Prenatal urinary BPA concentrations were not associated with increased BMI or waist circumference in these preschool-age children. In fact, consistent with the results from a prospective birth cohort study in California, we found that higher maternal urinary BPA concentrations were generally, but not significantly, associated with lower BMI in girls. Harley et al. (2013) reported modest decreases in child BMI at 9 years of age among girls born to women with the highest prenatal urinary BPA concentrations.

Our findings and those of Harley et al. (2013) are not consistent with those from a prospective cohort in Spain. Valvi et al. (2013) found that maternal urinary BPA concentrations during pregnancy were associated with increased child BMI and waist circumference at 4 years of age. They also reported that these associations were stronger among women who smoked during their pregnancy, but did not find any differences by child sex. Maternal smoking did not modify the association between prenatal BPA concentrations and child BMI in our cohort, but the statistical power to detect this modification was limited by the relatively small number of smokers.

Early-childhood urinary BPA concentrations were not associated with BMI or waist circumference at 2–5 years of age in this cohort, and point estimates were negative in direction. In contrast, two cross-sectional studies, one from California and another using the National Health and Nutrition Examination Survey, observed positive associations between urinary BPA concentrations and BMI or percent body fat among school-age and adolescent children (Harley et al. 2013; Trasande et al. 2012). Consistent with our findings, the New England Children’s Amalgam Trial, a randomized, prospective trial of dental amalgams and composite fillings did not find an association between childhood exposure to BPA–diglycidyl dimethacrylate composite dental fillings and increased child BMI or percent body fat (Maserejian et al. 2012).

With regard to the three prospective cohort studies (including the present study) with urinary BPA measurements, the differences in study design, urinary BPA concentrations, or the timing of BMI measurements do not seem to explain the discrepancies in their results. All three cohorts measured urinary BPA concentrations twice during pregnancy, and the two U.S. studies measured children’s BPA concentrations at the time BMI was assessed. Maternal urinary BPA concentrations were higher among women in this (median, 2.1 μg/L) and the Spanish (median, 2.1 μg/L) study than in the California study (median, 1.1 μg/L) (Harley et al. 2013; Valvi et al. 2013). Child BMI was measured longitudinally at 2–5 years of age in this study, at 14 months and 4 years in the Spanish study, and at 9 years of age in the California study. Confounding, BPA exposure misclassification, differences in distributions of effect measure modifiers, incorrect model specification, or residual sources of selection bias may explain the discrepancies across epidemiological studies (Howe et al. 2011).

Positive associations between urinary BPA concentrations and BMI or percent body fat in cross-sectional analyses may be attributable to residual confounding from unmeasured dietary sources of BPA exposure that are also important determinants of adiposity (e.g., soda or canned foods). It has also been suggested that BPA may simply be a marker of certain dietary patterns associated with obesity (Sharpe and Drake 2013). However, this hypothesis bears further scrutiny in light of the known dietary sources of BPA and the effect of these foods on obesity or cardiometabolic disease risk. For instance, canned foods are a major source of BPA in adults (Carwile et al. 2011; von Goetz et al. 2010), and some canned foods contain high levels of fiber and other micronutrients (e.g., canned beans), whereas others may be less nutritious (e.g., canned pasta). Thus, the direction of confounding will depend on the dietary source of BPA exposure and its association with obesity. Traditional measures of dietary quality, like food frequency questionnaires, may misspecify dietary confounding because these measures are designed to assess macro- or micronutrient intake rather than contaminants present in the food. Thus, there is a need to develop and control for dietary quality measures that incorporate potential sources of BPA exposure.

One strength of our study was the ability to control for socioeconomic, perinatal, and environmental factors, including tobacco smoke and phthalate exposures. However, we had imperfect measures of maternal and child diet and physical activity. Our results were not substantially different when we controlled for these measures of diet or physical activity; however, the inability to adjust for more accurate diet and activity measures may have biased our results.

Associations between urinary BPA concentrations and BMI may be attributable to physiological changes during pregnancy or early childhood that affect BPA excretion and fetal/child growth. For instance, children with BMI trajectories that have an early BMI nadir are at an increased risk of obesity or overweight compared with children who grow normally (Williams and Goulding 2009). Children on different growth trajectories may have different BPA or creatinine excretion patterns before they develop obesity, making it difficult to disentangle associations between urinary BPA concentrations and body composition from prodromal obesity-induced pharmacokinetic changes, even with prospective data. We speculate that this may be why the association between early-childhood urinary BPA concentrations and child BMI was attenuated when we did not adjust for creatinine. Although creatinine normalization is commonly used to account for urine dilution, it may not be an ideal marker of urine dilution when examining obesity or body composition.

It is well established that urinary BPA concentrations exhibit a high degree of within-person variability due to the relatively short biological half-life of BPA and the episodic nature of exposure (Braun et al. 2012; Teitelbaum et al. 2008; Volkel et al. 2002). This may lead to exposure misclassification, reduced statistical power for detecting potential associations, and null-attenuated effect estimates. This would substantially reduce our power to determine whether sex, race, prenatal tobacco smoke exposure, or maternal folate levels modified the association between urinary BPA concentrations and BMI. This is evident from the wider confidence intervals for the sex-specific associations compared with the full-cohort associations.

Another strength of our study is that we had up to four serial urinary BPA measures—two during pregnancy and two during early childhood—but even this may be insufficient to accurately classify exposure over long time periods. In addition, BPA exposure during narrower time windows or at other times of development (e.g., first trimester) may be more important than the exposure windows we measured. Alternative methods and matrices of BPA exposure assessment should be considered in future studies. Collecting more spot urine samples may improve exposure assessment, but the number of samples needed to ensure reasonable exposure classification and the impact of collecting these additional specimens on participants’ compliance is unknown. Other matrices that may have less within-person variability or reflect longer periods of exposure, such as meconium or shed deciduous teeth, should be considered and investigated when feasible.

We used up to four repeated BMI measures in children to quantify the association between early-life BPA exposures and absolute differences in child BMI, as well as the BMI slope between 2 and 5 years. Repeated BMI measures will reduce misclassification of child adiposity. However, child BMI is related to both fat and lean mass, and may not be highly correlated with direct measures of adiposity, especially when a child’s BMI is < the 85th percentile (Freedman et al. 2005). Other measures of body composition, including densitometry, bioelectric impedance, and dual energy X-ray absorptiometry can quantify fat and lean mass in separate body compartments to provide more accurate phenotyping of adipose distribution (Wells 2001). Future research should examine the relationship between BPA exposures and more refined body composition measures in children.

A prior rodent study found that dietary methyl donors (e.g., folic acid) might reduce obesity risk from prenatal BPA exposure (Dolinoy et al. 2007), but another did not (Rosenfeld et al. 2013). We did not find that the association between prenatal urinary BPA concentrations and child BMI differed according to maternal folate status. This may be attributable to differences in the timing of BPA or folate measurement in our study relative to dosing in animal studies, higher BPA exposure in animal studies compared with human exposures, or species-specific responses.

Prenatal and early-childhood BPA exposures were not associated with increased child BMI at 2–5 years of age in this birth cohort. There was some evidence suggesting that higher early-childhood BPA exposures were associated with faster BMI velocity, but this association was attenuated without creatinine adjustment. Further follow-up in this and larger cohort studies is warranted to assess whether prenatal or early-childhood BPA exposure ultimately results in higher BMI at later ages. In addition, studies in larger cohorts are needed to quantify potential sex-specific associations. Future studies should develop and integrate more refined measures of nutrient intake that incorporate potential sources of BPA exposure and improve methods to assess BPA exposure.

## Supplemental Material

(633 KB) PDF
