# Supplementary material for: Early-Life Bisphenol A Exposure and Child Body Mass Index: A Prospective Cohort Study
Source: Environ Health Perspect. 2014 Jul 29;122(11):1239–45. doi: 10.1289/ehp.1408258 (PMC4216170; doi:10.1289/ehp.1408258)
Supplement: (633 KB) PDF [file ehp.1408258.s001.508.pdf]

Supplemental Material

Early-Life Bisphenol A Exposure and Child Body Mass Index: A Prospective Cohort Study

Joseph M. Braun, Bruce P. Lanphear, Antonia M. Calafat, Sirad Deria, Jane Khoury, Chanelle J. Howe, and Scott A. Venners

Table of Contents **Page**

Table S1. Timing of urine sample collection in pregnant women and their children from Cincinnati, OH (2003-2012) 2

Table S2. Urinary BPA concentrations during pregnancy (2003-2006) and the 1st 5 years of life (2004-2012) among Cincinnati, OH women and their children 3

Table S3. Pearson correlation coefficients between $\log_{10}$-transformed urinary BPA concentrations in Cincinnati, OH women and their children 4

Table S4. Unadjusted and adjusted change in child BMI z-score between 2-5 years of age with a 10-fold increase in maternal or early childhood urinary BPA concentrations 5

Table S5. Sensitivity analyses examining robustness of results to various exclusions and analytic assumptions 6

Table S6. Adjusted change in child waist circumference between 4-5 years of age by tercile of or with a 10-fold increase in maternal or early childhood urinary BPA concentrations 7

Table S7. Adjusted change in child BMI z-score between 2-5 years of age with a 10-fold increase in maternal or early childhood urinary BPA concentrations-stratified by child sex 8

Table S8. Adjusted cross-sectional associations between child urinary BPA concentrations and BMI z-scores at 2-5 years of age 9

Figure S1. Distribution of HOME Study children's BMI z-scores at 2, 3, 4, and 5 years of age 10

Figure S2. Adjusted BMI z-score slopes between 2 and 5 years of age by prenatal urinary BPA concentration and child sex 11

Figure S3. Adjusted BMI z-score slopes between 2 and 5 years of age by early childhood urinary BPA concentration and child sex 13

Table S1. Timing of urine sample collection in pregnant women and their children from Cincinnati, OH (2003-2012).

Measurement	N	Mean	SD	Min	Max
16 Week	296	16.0	1.9	11.3	22.6
26 Week	290	26.5	2.0	21.6	34.6
1 Year	249	1.08	0.09	0.94	1.61
2 Year	231	2.09	0.08	1.84	2.38
3 Year	231	3.10	0.12	2.88	3.62
4 Year	168	4.13	0.13	3.77	4.65
5 Year	199	5.20	0.24	4.75	6.00

16 and 26 week values are units of gestational week. 1-5 year values are in units of years.

Table S2. Urinary BPA concentrations during pregnancy (2003-2006) and the 1st 5 years of life (2004-2012) among Cincinnati, OH women and their children.

Creatinine adjustment and time of urine measurement	N	p5	p25	p50	p75	p95
**Non-standardized (mg/L)**						
Average prenatal	297	0.4	1.1	2.1	3.9	11.4
16 Week	297	0.3	0.9	2.0	3.3	11.4
26 Week	290	0.3	0.8	1.8	3.3	10.4
Average 1+2 year	287	0.8	1.8	3.6	6.9	16.1
1 Year	251	0.5	1.7	3.8	7.3	21.6
2 Year	233	0.6	1.5	2.9	7.4	24.3
3 Year	233	0.5	1.4	2.9	5.1	16.4
4 Year	170	0.3	1.1	2.0	4.1	14.4
5 Year	201	0.5	1.1	2.3	3.7	11.7
**Creatinine standardized (mg/g creatinine)**						
Average prenatal	297	0.9	1.5	2.1	3.5	8.8
16 Week	296	0.6	1.1	1.8	3.0	10.7
26 Week	290	0.8	1.4	2.0	3.3	8.1
Average 1+2 year	285	4.7	8.4	13.7	23.6	54.5
1 Year	249	5.1	10.0	17.9	32.5	72.7
2 Year	231	3.3	5.9	10.5	18.3	65.0
3 Year	231	1.5	3.1	5.3	9.4	23.2
4 Year	168	1.2	2.3	3.7	6.1	15.4
5 Year	199	1.2	1.9	3.1	5.3	16.2

Number of mothers/children for the average concentrations is greater than the individual concentrations since not all the same participants returned at the both visits.

Table S3. Pearson correlation coefficients between $\log_{10}$-transformed urinary BPA concentrations in Cincinnati, OH women and their children.

Creatinine adjustment and BPA exposure measure	Maternal: average	Maternal: 16 week	Maternal: 26 week	Child: average	Child: 1 year	Child: 2 year
**No creatinine-normalization**						
Maternal: average	1.00	0.81*	0.81*	0.17*	0.12	0.19*
Maternal: 16 week		1.00	0.30*	0.12*	0.04	0.15*
Maternal: 26 week			1.00	0.15*	0.15*	0.17*
Child: average				1.00	0.81*	0.83*
Child: 1 year					1.00	0.19*
Child: 2 year						1.00
**Creatinine-normalized**						
Maternal: average	1.00	0.77*	0.72*	0.03	0.10	-0.05
Maternal: 16 week		1.00	0.09	0.03	0.01	0.01
Maternal: 26 week			1.00	0.01	0.14*	-0.07
Child: average				1.00	0.78*	0.80*
Child: 1 year					1.00	0.10
Child: 2 year						1.00

*Indicates that Pearson correlation is statistically significant ($p < 0.05$).

Table S4. Unadjusted and adjusted change in child BMI z-score between 2-5 years of age with a 10-fold increase in maternal or early childhood urinary BPA concentrations.

Adjustments	Prenatal beta (CI)	Prenatal p-value	Early childhood beta (CI)	Early childhood p-value
Unadjusted	-0.2 (-0.7, 0.2)	0.24	-0.1 (-0.5, 0.2)	0.42
SES + Perinatal variables[a]	-0.1 (-0.5, 0.3)	0.51	-0.2 (-0.6, 0.1)	0.19
SES + Perinatal + Maternal Nutrition[b]	-0.1 (-0.5, 0.3)	0.48	-0.2 (-0.5, 0.1)	0.24
SES + Perinatal + Child Nutrition[c]	-0.1 (-0.5, 0.3)	0.57	-0.2 (-0.6, 0.1)	0.20
SES + Perinatal + Child Activity[d]	-0.1 (-0.5, 0.3)	0.54	-0.2 (-0.6, 0.1)	0.19
SES + Perinatal + Child Activity + Maternal and Child Nutrition	-0.1 (-0.5, 0.3)	0.54	-0.2 (-0.5, 0.1)	0.24
SES + Perinatal + Child Activity + Maternal and Child Nutrition + Pre- and Postnatal BPA	-0.2 (-0.6, 0.3)	0.45	-0.2 (-0.5, 0.1)	0.24
SES + Perinatal + Child Activity + Maternal and Child Nutrition + Pre- or Postnatal urinary DEHP[e]	-0.1 (-0.5, 0.3)	0.58	-0.2 (-0.6, 0.1)	0.23
SES + Perinatal + Child Activity + Maternal and Child Nutrition + Child Age[f]	-0.1 (-0.5, 0.3)	0.48	-0.2 (-0.6, 0.1)	0.20
Adjusted for postnatal cotinine	-0.1 (-0.6, 0.3)	0.49	-0.2 (-0.6, 0.1)	0.25

[a]Maternal race (white, black, and other), marital status (married living together, unmarried living together, and unmarried living alone), parity (0, 1, and 2+), age at delivery (continuous in years), household income (continuous in $10,000 increments), education (< high school, high school, some college, and ≥ bachelor's degree), employment (any and none), insurance (private and public/none), BMI at 16 weeks (continuous in kg/m^2), depressive symptoms at baseline (continuous), and prenatal serum cotinine (continuous, $\log_{10}$-transformed). [b]Prenatal vitamin intake (weekly/daily and rarely/never), prenatal canned vegetable consumption (≥ daily, weekly, and ≤ monthly), and prenatal fresh fruit/vegetable intake (monthly/weekly vs. ≥ daily). [c]Child canned vegetable consumption (≥ daily, weekly, and ≤ monthly), child fresh fruit/vegetable intake (monthly/weekly vs. ≥ daily), and duration of any breastfeeding (weeks). [d]Number of hours child watches television or DVD/videos (continuous in hours) and number of hours child plays outside (continuous in hours). [e]Prenatal models adjusted for average maternal urinary di-2-ethylhexyl phthalate (DEHP) levels during pregnancy. Postnatal models adjusted for average child urinary DEHP levels during the first two years of life. [f]Child age in months at time of BMI measurement.

Table S5. Sensitivity analyses examining robustness of results to various exclusions and analytic assumptions.

Adjustments	Prenatal beta (CI)	Prenatal p-value	Early childhood beta (CI)	Early childhood p-value
Unadjusted	-0.2 (-0.7, 0.2)	0.24	-0.1 (-0.5, 0.2)	0.42
Adjusted[a]	-0.1 (-0.5, 0.3)	0.51	-0.2 (-0.6, 0.1)	0.19
Adjusted-No SGA[a,b]	-0.3 (-0.6, 0.1)	0.22	-0.1 (-0.4, 0.2)	0.51
Adjusted-No GDM[a,b]	-0.1 (-0.5, 0.3)	0.64	-0.2 (-0.6, 0.1)	0.15
Adjusted-No PIH[a,b]	-0.2 (-0.6, 0.3)	0.47	-0.3 (-0.7, 0.1)	0.10
Adjusted-exclude high prenatal BPA value[a]	-0.1 (-0.5, 0.4)	0.76	-0.2 (-0.6, 0.1)	0.17
Adjusted-No creatinine normalization[a]	-0.1 (-0.6, 0.3)	0.49	-0.2 (-0.6, 0.1)	0.25

[a]Maternal race (white, black, and other), marital status (married living together, unmarried living together, and unmarried living alone), parity (0, 1, and 2+), age at delivery (continuous in years), household income (continuous in \$10,000 increments), education (< high school, high school, some college, and ≥ bachelor's degree), employment (any and none), insurance (private and public/none), BMI at 16 weeks (continuous in kg/m^2), depressive symptoms at baseline (continuous), and prenatal serum cotinine (continuous, log_{10}-transformed). [b]Small for gestational age (SGA, n = 24), gestational diabetes mellitus (GDM, n = 13), and pregnancy induced hypertension (PIH, n = 29).

Beta represents change in child BMI z-score with a 10-fold increase in child urinary BPA concentrations.

Table S6. Adjusted change in child waist circumference between 4-5 years of age by tercile of or with a 10-fold increase in maternal or early childhood urinary BPA concentrations.[a]

BPA exposure measure	N	Mean waist circumference (cm)	Beta (CI)	p-value
**Prenatal**				
1st Tercile (0.4-1.6 mg/g Cr)	70	52.5	Ref	
2nd Tercile (1.6-2.6 mg/g Cr)	71	51.9	-0.5 (-2.0, 0.9)	0.45
3rd Tercile (2.7-49 mg/g Cr)	70	52.0	-0.5 (-2.3, 1.3)	0.60
Continuous, log_{10}-transformed	211		-1.5 (-4.0, 1.0)	0.24
**Early childhood**				
1st Tercile (3.2-11 mg/g Cr)	67	52.5	Ref	
2nd Tercile (11-20 mg/g Cr)	68	52.4	-0.1 (-1.5, 1.3)	0.94
3rd Tercile (20-233 mg/g Cr)	67	51.5	-1.0 (-2.5, 0.6)	0.22
Continuous, log_{10}-transformed	202		-1.2 (-3.4, 1.0)	0.28

[a]Maternal race (white, black, and other), marital status (married living together, unmarried living together, and unmarried living alone), parity (0, 1, and 2+), age at delivery (continuous in years), household income (continuous in $10,000 increments), education (< high school, high school, some college, and ≥ bachelor's degree), employment (any and none), insurance (private and public/none), BMI at 16 weeks (continuous in kg/m^2), depressive symptoms at baseline (continuous), and prenatal serum cotinine (continuous, log_{10}-transformed).

Table S7. Adjusted change in child BMI z-score between 2-5 years of age with a 10-fold increase in maternal or early childhood urinary BPA concentrations-stratified by child sex.[a]

Creatinine adjustment and child sex strata	Prenatal beta (CI)	Prenatal p-value	Early childhood beta (CI)	Early childhood p-value
**Creatinine normalized**				
Girls	-0.4 (-0.9, 0.2)	0.21	-0.6 (-1.1, -0.1)	0.02
Boys	0.0 (-0.5, 0.6)	0.88	0.1 (-0.4, 0.5)	0.80
**Not creatinine normalized**				
Girls	-0.1 (-0.5, 0.2)	0.44	-0.2 (-0.6, 0.1)	0.22
Boys	0.1 (-0.4, 0.5)	0.71	0.2 (-0.1, 0.6)	0.14

[a]Maternal race (white, black, and other), marital status (married living together, unmarried living together, and unmarried living alone), parity (0, 1, and 2+), age at delivery (continuous in years), household income (continuous in $10,000 increments), education (< high school, high school, some college, and ≥ bachelor's degree), employment (any and none), insurance (private and public/none), BMI at 16 weeks (continuous in kg/m^2), depressive symptoms at baseline (continuous), and prenatal serum cotinine (continuous, log_{10}-transformed).

Child sex effect measure modification (EMM) p-value for prenatal, creatinine-normalized = 0.30, EMM p-value for prenatal without creatinine-normalization = 0.39, EMM p-value for early childhood creatinine-normalized = 0.05, and EMM p-value for early childhood without creatinine-normalization = 0.06.

Table S8. Adjusted cross-sectional associations between child urinary BPA concentrations and BMI z-scores at 2-5 years of age.[a]

**Child age**	**N**	**Beta (CI)**	**p-Value**
2 Year	226	-0.1 (-0.4, 0.2)	0.66
3 Year	217	0.2 (-0.2, 0.7)	0.27
4 Year	167	0.1 (-0.3, 0.6)	0.63
5 Year	199	0.0 (-0.3, 0.4)	0.82

[a]Maternal race (white, black, and other), marital status (married living together, unmarried living together, and unmarried living alone), parity (0, 1, and 2+), age at delivery (continuous in years), household income (continuous in $10,000 increments), education (< high school, high school, some college, and ≥ bachelor's degree), employment (any and none), insurance (private and public/none), BMI at 16 weeks (continuous in kg/m^2), depressive symptoms at baseline (continuous), and prenatal serum cotinine (continuous, log_{10}-transformed).

Beta represents change in child BMI z-score with a 10-fold increase in child urinary BPA concentrations.

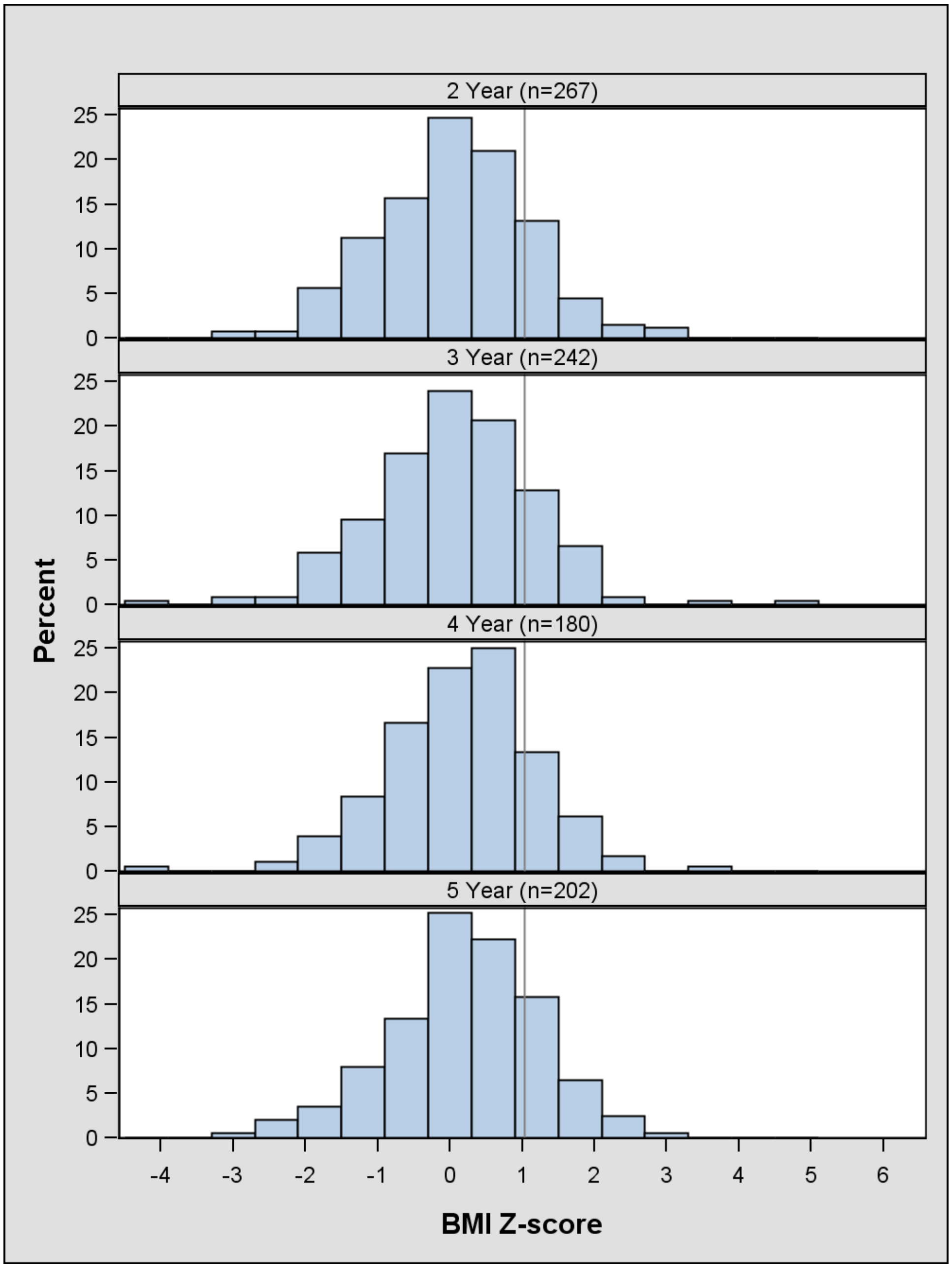

Figure S1. Distribution of HOME Study children's BMI z-scores at 2, 3, 4, and 5 years of age. Grey line represents the 85th percentile of BMI z-score, the threshold for being overweight.

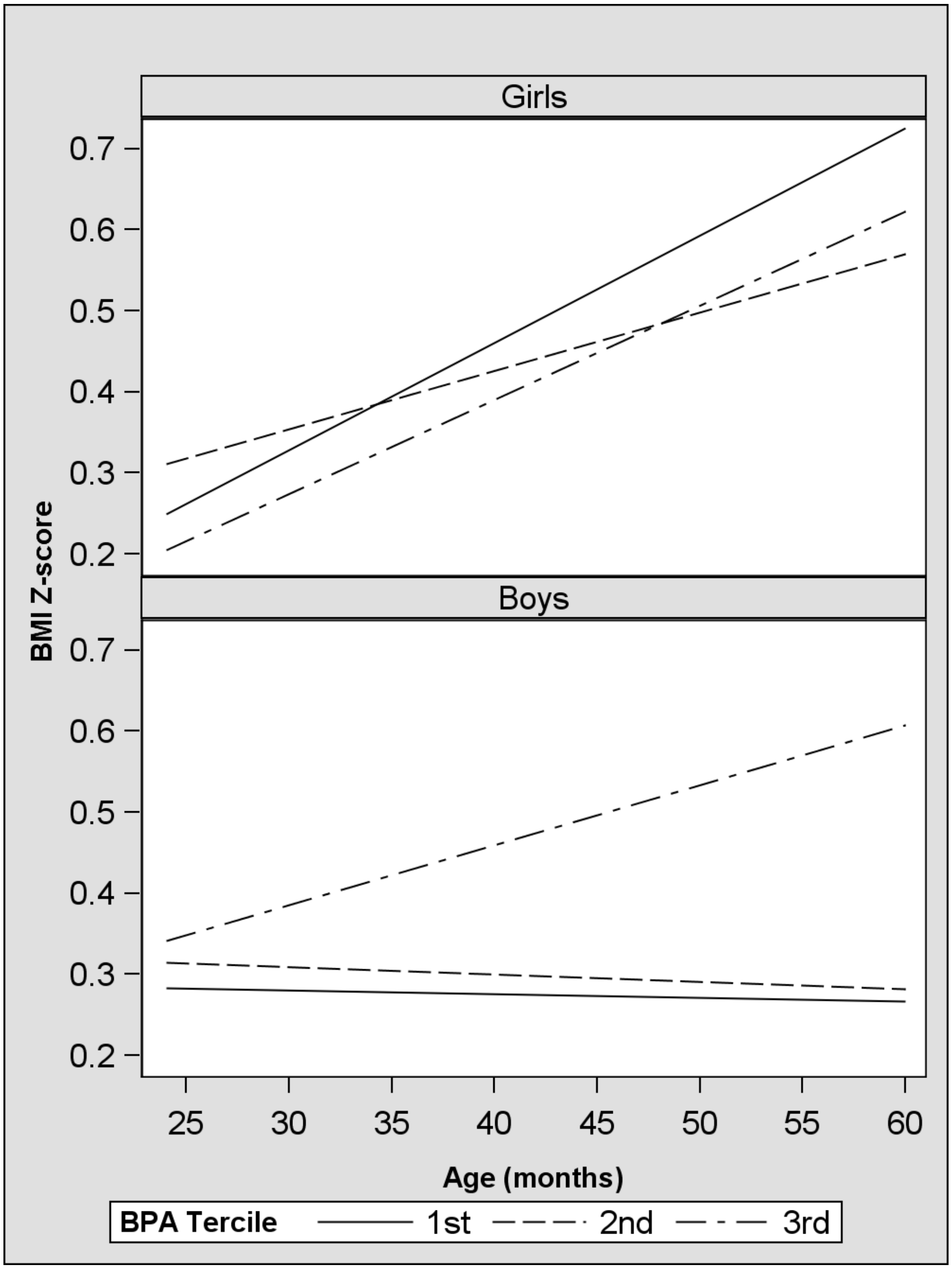

Figure S2. Adjusted BMI z-score slopes between 2 and 5 years of age by prenatal urinary BPA concentration and child sex. Adjusted for maternal race (white, black, and other), marital status (married living together, unmarried living together, and unmarried living alone), parity (0, 1, and 2+), age at delivery (continuous in years), household income (continuous in $10,000 increments), education (< high school, high school, some college, and ≥ bachelor's degree), employment (any and none), insurance (private and public/none), BMI at 16 weeks (continuous in kg/m^2),

depressive symptoms at baseline (continuous), and prenatal serum cotinine (continuous, $\log_{10}$-transformed). The BMI slope was greatest among boys in the 3rd BPA tercile (slope = 0.09, 95% CI: -0.01, 0.19) compared to boys in the 1st (slope = -0.01, 95% CI: -0.08, 0.07) and 2nd tercile (slope = -0.01, 95% CI: -0.08, 0.06) BPA terciles (age x BPA effect measure modification p = 0.13). The BMI slopes were not different among girls in the 1st (slope = 0.16, 95% CI: 0.06, 0.25), 2nd (slope = 0.09, 95% CI: -0.01, 0.18), or 3rd (slope = 0.14, 95% CI: 0.07, 0.21) BPA terciles (age x BPA effect measure modification p = 0.77).

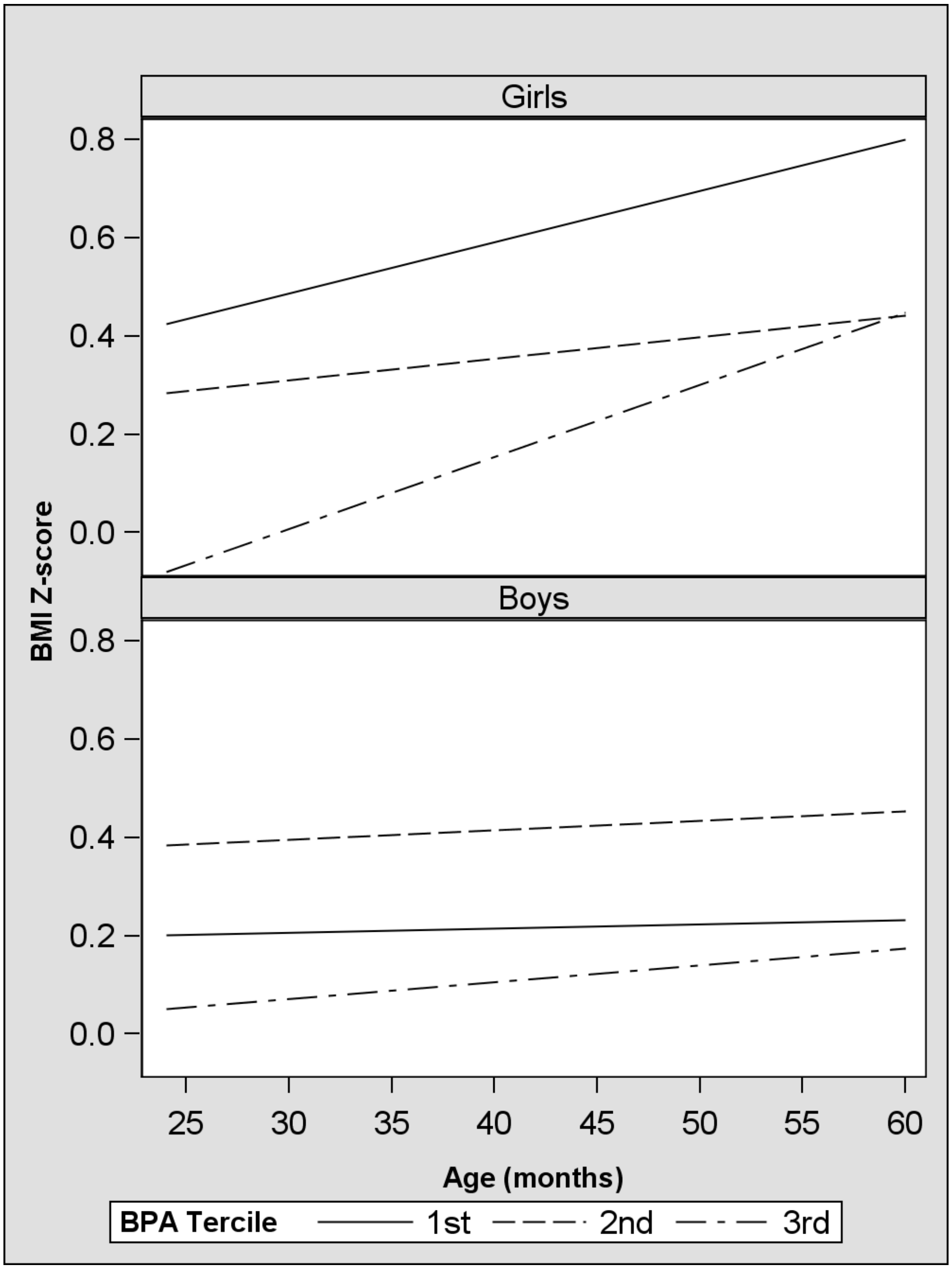

Figure S3. Adjusted BMI z-score slopes between 2 and 5 years of age by early childhood urinary BPA concentration and child sex. Adjusted for maternal race (white, black, and other), marital status (married living together, unmarried living together, and unmarried living alone), parity (0, 1, and 2+), age at delivery (continuous in years), household income (continuous in $10,000 increments), education (< high school, high school, some college, and ≥ bachelor's degree), employment (any and none), insurance (private and public/none), BMI at 16 weeks

(continuous in kg/m^2), depressive symptoms at baseline (continuous), and prenatal serum cotinine (continuous, log_{10}-transformed). The BMI slope was lowest among girls 2nd (slope = 0.05, 95% CI: -0.03, 0.14) BPA tercile and highest among girls in the 1st (slope = 0.12, 95% CI: 0.05, 0.20) and 3rd (slope = 0.18, 95% CI: 0.10, 0.25) BPA terciles (age x BPA effect measure modification p = 0.28). The BMI slopes were not different among boys in the 1st (slope = 0.01, 95% CI: -0.09, 0.11), 2nd (slope = 0.02, 95% CI: -0.06, 0.10), or 3rd (slope = 0.04, 95% CI: -0.02, 0.10) BPA terciles (age x BPA effect measure modification p = 0.62).
